# Dynamic susceptibility contrast MRI may contribute in prediction of stereotactic radiosurgery outcome in brain metastases

**DOI:** 10.1093/noajnl/vdac070

**Published:** 2022-05-13

**Authors:** Lea Starck, Bente Sandvei Skeie, Gunnar Moen, Renate Grüner

**Affiliations:** Department of Physics and Technology, University of Bergen, Bergen, Norway; Department of Radiology, Mohn Medical Imaging and Visualization Centre, Haukeland University Hospital, Bergen, Norway; Department of Neurosurgery, Haukeland University Hospital, Bergen, Norway; Department of Radiology, Haukeland University Hospital, Bergen, Norway; Department of Physics and Technology, University of Bergen, Bergen, Norway; Department of Radiology, Mohn Medical Imaging and Visualization Centre, Haukeland University Hospital, Bergen, Norway; Department of Radiology, Haukeland University Hospital, Bergen, Norway

**Keywords:** brain metastases, DSC-MRI, perfusion, pseudo-progression, stereotactic radiosurgery

## Abstract

**Background:**

Following stereotactic radiosurgery (SRS), predicting treatment response is not possible at an early stage using structural imaging alone. Hence, the current study aims at investigating whether dynamic susceptibility contrast (DSC)-MRI estimated prior to SRS can provide predictive biomarkers in response to SRS treatment and characterize vascular characteristics of pseudo-progression.

**Methods:**

In this retrospective study, perfusion-weighted DSC-MRI image data acquired with a temporal resolution of 1.45 seconds were collected from 41 patients suffering from brain metastases. Outcome was defined based on lesion volume changes in time (determined on structural images) or death. Motion correction and manual lesion delineation were performed prior to semi-automated, voxel-wise perfusion analysis. Statistical testing was performed using linear regression and a significance threshold at *P* = .05. Age, sex, primary cancers (pulmonary cancer and melanoma), lesion volume, and dichotomized survival time were added as covariates in the linear regression models (ANOVA).

**Results:**

Relative cerebral blood volume (rCBV) and relative cerebral blood flow (rCBF) were found to be significantly lower prior to SRS treatment in patients with increasing lesion volume or early death post-SRS (*P* ≤ .01).

**Conclusion:**

Unfavorable treatment outcome may be linked to low perfusion prior to SRS. Pseudo-progression may be preceded by a transient rCBF increase post-SRS. However, results should be verified in different or larger patient material.

**Key Points**

- Non-progressing brain metastases patients have lower rCBF and rCBV prior to SRS than progressors or short survivors.
- Pseudo-progression may be preceded by a transient rCBF increase post-SRS.

**Importance of the Study**

Incidence of cancer patients with brain metastases range between 5.6% and 9.6%, and detection techniques are improving. Stereotactic radiosurgery is the primary treatment strategy for brain metastases. Physiological markers connected to treatment response, ie, tumor progression, non-progression, and survival could become important for treatment decisions and disease monitoring, particularly if preceding structural findings. Our results suggest that low dynamic susceptibility contrast MRI-derived perfusion parameters prior to stereotactic radiosurgery may increase the probability of poor treatment response.

Globally, 5-year overall cancer survival is increasing^1^ and brain metastasis detection techniques have improved substantially.^2^ Approximately 1.8 million new cancer cases were estimated in the United States in 2019.^3^ Reports on brain metastases incidence vary, with population studies reporting percentages of cancer patients with brain metastases ranging from 5.6% to 9.6%.^4–6^ Stereotactic radiosurgery (SRS) is recommended in the clinic,^7–9^ and is the current primary treatment strategy, though synergic effects with other strategies are being investigated.^10,11^

An apparent volumetric growth with tissue enhancement in structural MR images post-treatment (ie, SRS, radiotherapy, chemotherapy, or combinations thereof) does not necessarily signify progressive disease.^12,13^ The increased tissue contrast enhancement can be a sign of disease progression, but it can also be a sign of accumulation of fluids in the radiation-induced scar tissue or local tissue inflammation.^12–14^ The latter scenario is an example of pseudo-progression. Pseudo-progression has been reported in as many as 32% of cases of apparent volumetric growth in brain metastases.^15^ Since distinguishing progression from pseudo-progression using structural images alone is near impossible at an early stage,^12,16,17^ possible pseudo-progression complicates the development of predictive biomarkers of response to treatment. This is also reflected by the continued research to determine the level of metastatic progression with other imaging modalities as well.^12,14,18^ These modalities include diffusion MRI,^19^ dynamic contrast-enhanced (DCE)-MRI,^20,21^ MR spectroscopy,^22,23^ positron emission tomography approaches,^24^ and volume progression modeling.^23^ The role of microvascular perfusion as measured by dynamic susceptibility contrast (DSC)-MRI in predicting progression vs pseudo-progression outcome in primary brain cancers,^25,26^ as well as in brain metastases,^22–24,27–29^ has also been investigated. In general, high relative cerebral blood volume (rCBV) post-treatment is associated with poorer outcome,^22–29^ though not all reported clinical studies have been able to reproduce these results.^30^ Decreases in rCBV between baseline and the follow-up scans have been reported to be sensitive to pseudo-progression after SRS.^21^

Though pseudo-progression may hamper treatment response predictions in the clinic, perfusion parameters can be investigated as predictive biomarkers of treatment response. In a cross-sectional study, 1-month post-follow-up, it was shown that brain metastatic disease progressors had lower rCBV compared to treatment responders.^20^ Local recurrence has been associated with higher rCBV.^31^ Reduced relative cerebral blood flow (rCBF) compared to baseline at 6-week follow-up has been reported to predict treatment response.^32^ However, none of these results were based on data from pre-SRS treatment, ie, baseline perfusion data. Using true baseline data, one study reported improved vascularization of the peritumoral tissue in treatment responders compared to pseudo-progressors,^33^ and high rCBV prior to radiotherapy was identified as a predictor for survival in glioma.^34^ Others did not find that pretherapeutic rCBV and rCBF correlated with treatment outcome.^32,35^

The aim of the current study is to investigate whether perfusion estimates based on DSC-MRI prior to SRS (baseline) differentiate between patients with good treatment outcome, ie, brain metastases are reduced in volume or exhibit a behavior commonly seen in pseudo-progression (transient volume increase post-SRS) from patients with poor prognosis. The latter group included patients with consistent volume increase post-SRS or missing follow-up visits due to early end-of-life (EOF). Average perfusion estimated across the enhancing lesion volumes is also analyzed and compared both across patients (baseline) and in time (ie, in patients with longer survival) in order to investigate similarities in perfusion characteristics across patients.

## Methods

### Data Collection

**Figure 1.**
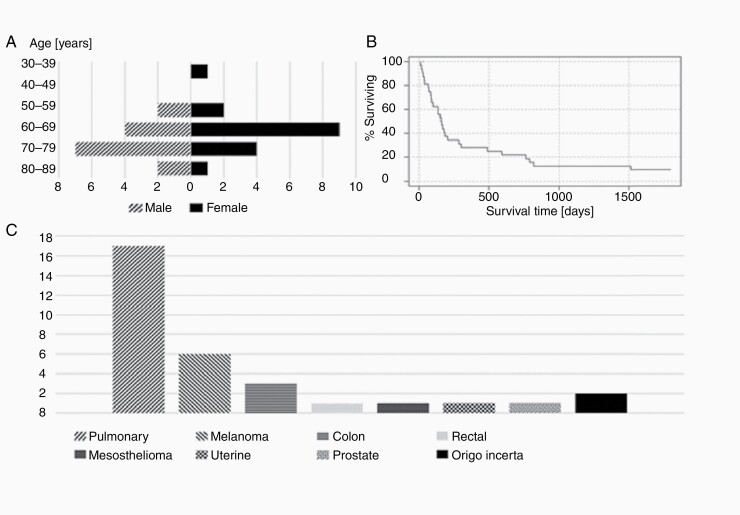
Patient cohort description. Age distribution (years) of imaged male and female patients (A), percentage survival time (B), and tumor origin (C) in the 32 patients included in the analysis.

Between 2010 and 2015, longitudinal MRI data were collected in 41 adult patients with brain metastases of heterogeneous origin. 32 patients (15 males, 17 females) with a mean and median age of 67 and 68 years, respectively, met the inclusion criteria with metastases ≥0.50 cc at baseline. A comprehensive imaging protocol including structural T_1_-weighted (T_1_w) and perfusion-weighted images using gadolinium-based contrast agent, was acquired immediately prior to frame fixation for SRS treatment. Eleven patients had multiple metastases, but only the single largest metastasis at baseline was included in the analysis in the current study. All imaging was performed at the same treatment site, which serves as a national gamma knife SRS treatment facility. The first treatment follow-up MRI scan was performed 1 month after SRS, subsequently at intervals of 3 months. The study was performed according to guidelines and with ethical approval of the regional committee for health and research ethics. Informed consent was given by all patients. In addition to MR scans, other clinical data were recorded. This included information about age, sex, tumor origin, and survival (Figure 1). Two patients received whole-brain radiotherapy (WBRT) before SRS and one patient had a craniotomy. 15 patients received chemotherapy.

### Imaging Protocol

A Siemens Symphony Vision 1.5 T whole-body MRI system (Siemens Erlangen, Germany) with an 8-channel head coil was used in all imaging. Acquired images include structural T_1_w images (2D spin echo, TR/TE/FA = 430 ms/9.4 ms/90°, slice thickness 5.5 mm, slice spacing 7.15 mm, imaging matrix 512 × 512 × 12, and 230 × 230 mm^2^ field of view) and co-localized perfusion-weighted DSC-MRI images (2D gradient recalled echo echo planar imaging (GRE-EPI), TR/TE/FA = 1450 ms/47 ms/90°, slice thickness 5.5 mm, slice spacing 7.15 mm, imaging matrix 128 × 128 × 12, and 230 × 230 mm^2^ field of view). With a temporal resolution of 1.45 seconds, a total of 60 volumes in time were collected during the DSC-MRI acquisition. The contrast agent Dotarem (Guerbet, Villepinte, France) was injected according to body weight (0.1 mmol/kg) using a power injector 10 seconds after acquisition start and with 5 mL/s injection speed. In addition, 3D T1 structural acquisition was performed based on the already injected contrast agent, either later in the same imaging session (follow-up visits) or after a short break in the MRI acquisition used for mounting the SRS treatment frame (baseline).

### Image Registration and Segmentation

Image registration of the pre- and post-contrast T_1_w images was performed with the command line tool *Elastix*,^36,37^ facilitating comparisons of various registration parameters. Providing the lower squared differences sum between voxels from the pre- and post-contrast images, an affine volume registration scheme was chosen.

Segmentation was performed by first subtracting pre-contrast T_1_w images from post-contrast T_1_w images.^38,39^ The different image was skull stripped with the FMRIB Software Library (FSL).^40^ To remove noise and over-enhancing areas (arteries) all pixels with values between 5% and 80% of maximum pixel value were set to one, whereas all others were set to zero. This ensuing binary mask was then overlaid the T_1_w post-contrast structural image to guide manual segmentation of the total lesion volume, providing masks covering the entire lesion. Enhancing lesion volumes were found by multiplying the total lesion volume maps by the binary mask, providing an ROI (region of interest) for extraction of mean rCBV and rCBF

### Perfusion Analysis

In DSC-MRI, the measured MR signal is dependent on the concentration of exogenous contrast agent in the volume of interest. The DSC-MRI perfusion parameters rCBV and rCBF are calculated based on measurements of the concentration curves. rCBV is the ratio of total contrast flowing through the voxel of interest divided by the total contrast flowing through a voxel ideally containing only blood in the same the ROI.

Calculating rCBF requires the deconvolution of the inflowing contrast concentration curve and the residue function. The residue function is a measure of the relative amount of contrast left in the voxel of interest after time *t*.

Parametric perfusion maps were estimated using NordicICE (NordicNeuroLab Inc, Bergen, Norway). Deconvolution was performed using singular value decomposition.^41^ Leakage effects were corrected for through a residue function-based leakage correction method, where the leakage was parametrically modeled.^42^ The 1% highest perfusion values were removed from the outputted parametric maps (ie, rCBV, rCBF maps) to remove effects attributed to flow turbulence.

**Figure 2.**
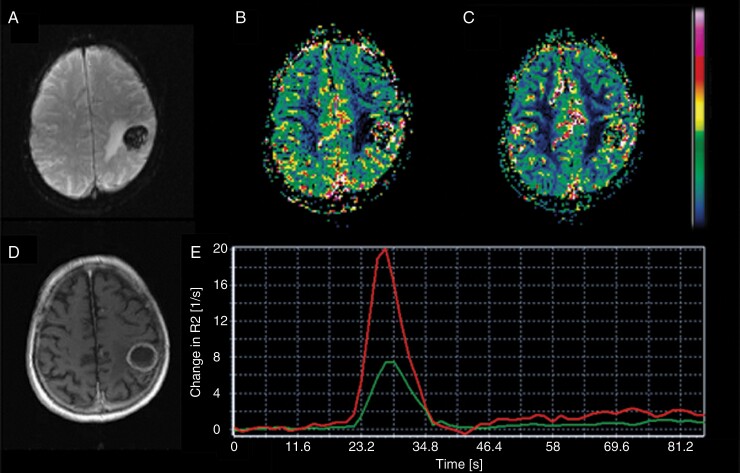
Perfusion analysis. Perfusion-weighted image (A), cerebral blood volume map (B), cerebral blood flow map (C), and structural T_1_-weighted image (D) in a randomly selected patient. The arterial input function (curve with higher peak) and a tissue response function (curve with lower peak) are also shown (E).

Perfusion maps were computed using a scan-specific semi-automated arterial input function (AIF). A search region was defined over the circle of Willis in each imaging session. In this search region, the NordicICE software detected five possible AIFs candidates by way of cluster analysis,^43^ applying measures of similarity typical to an AIF. The software thus chose voxels exhibiting contrast time curves with large area under the curve, a low baseline, and early, high peak enhancement. In each scan, the five AIF candidates were verified by visual inspection and averaged to yield the final AIFs. An example of the selected AIF in a randomly selected patient is displayed in Figure 2.

The low-resolution parametric perfusion maps were re-gridded to match the image pixel numbers of the structural T_1_w images, using MATLAB R2019b (MathWorks Inc., Natick, MA, USA). The parametric maps were then registered slice by slice to the structural contrast images with a 2D rigid translational registration. This way, a voxel-wise multiplication with the previously described lesion masks and enhancing masks could be performed, and the average perfusion parameter values within them calculated.

### Defining Progression and Non-Progression

Progressing and non-progressing metastatic tumor status was decided based on the volume of the metastatic lesion areas recorded in T_1_w follow-up scans. Increase and decrease were defined as at least 15% volume change relative to the previous volume. Only one metastasis showed no volumetric change according to this definition, and this was counted as non-progression. Death prior to the first possible follow-up or increase in metastatic lesion volume prior to a second SRS was interpreted as progression. Decrease in metastatic lesion volume was interpreted as non-progression, an initial increase in metastatic lesion volume, followed by a decrease prior to second SRS or death was interpreted as non-progression with pseudo-progression. During a lifetime follow-up, patients can in principle undergo both progression and non-progression. If both progression and pseudo-progression occurred in time, the patient was labeled according to whichever of the two events occurred first and prior to any second SRS.

### Statistical Testing

Comparison of perfusion parameters between patients experiencing progression and patients experiencing non-progression was performed using linear regression. Age, sex, lesion volume, frequent primary cancers (pulmonary cancer and melanoma) and dichotomized survival with a cut-off value at 60 days were considered as covariates. Linear regression models were systematically built by using all possible combinations of covariates. Each alternative model was compared to the non-corrected model using ANOVA testing. Receiver operator characteristic (ROC) curves were computed by fitting a logistic regression model to labeled progressors and non-progressors to help evaluate the predictive power of the perfusion parameters. Comparison of perfusion parameters between patients surviving for less than 60 days and patients surviving for more than 60 days was performed using linear regression. An unpaired two-sample *t* test was applied to test for group differences in patients that received chemotherapy vs those that did not, and to test whether patients with pulmonary metastases had longer or shorter survival than the remaining patients. All significance thresholds were set to *P* = .05.

## Results

Patients were classified as either progressors or non-progressors based on post-treatment volume changes. 12 patients were classified as progressors of which 5 patients showed immediate progression in the first follow-up after SRS and 7 patients showed delayed progression, ie, volumetric increase was not yet visible in the structural image data acquired at the first follow-up. 20 patients were identified as non-progressors, of which 13 patients were regressors and 7 patients were non-progressors but with pseudo-progression. All three patients that received WBRT, or craniotomy, were long survivors defined as non-progressors. In total, 14 out of 15 patients that received chemotherapy were defined as long survivors, and 13 were defined as non-progressors.

**Figure 3.**
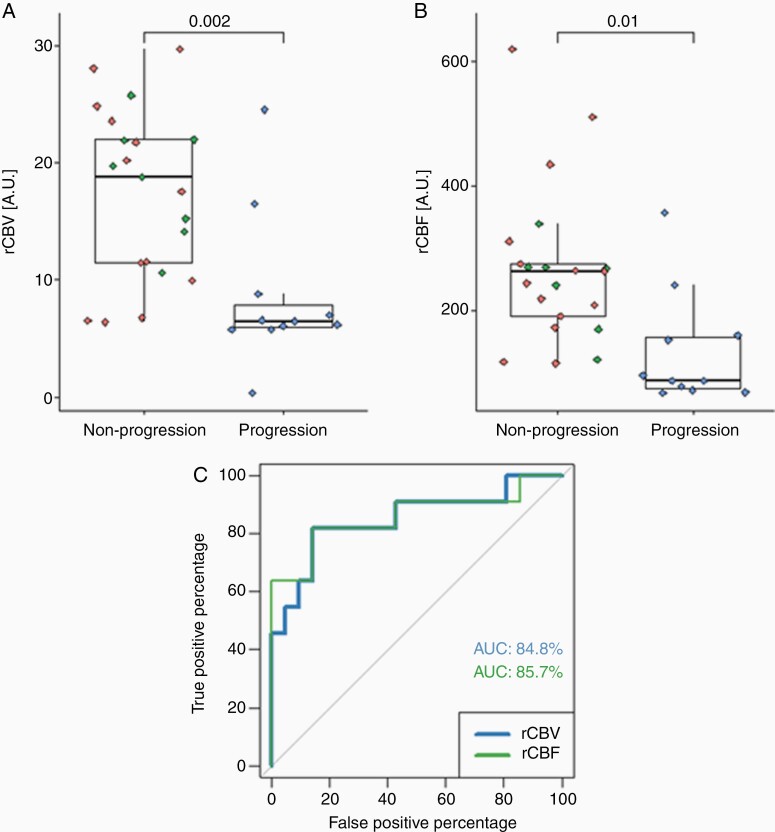
Distribution of relative cerebral blood flow (rCBV) (A) and relative cerebral blood volume (rCBF) (B) in tumor volume prior to stereotactic surgery in patients with tumor progression and patients with non-progression. The green dots among patients experiencing non-progression correspond to patients that also experienced pseudo-progression. No covariates were incorporated in the model for rCBV, but lesion volume and melanoma primary cancer were incorporated in the model for rCBF The *P*-values were calculated with linear regression models, and significance was defined at *P* < .05. Receiver operator characteristic (ROC) curves were computed to evaluate the accuracy of rCBF and rCBV as predictors across patients (C).

rCBV and rCBF were calculated within the ROI masks covering the enhancing metastases on the image data acquired the same day and prior to the SRS treatment. There was a significant difference between rCBV and rCBF from enhancing tumor area in progressors and non-progressors (Figure 3A and B). An ROC curve shows an AUC of 84.8% and 85.7 %, when rCBV and rCBF are used as predictors, respectively.

All possible combinations of age, sex, primary cancers (pulmonary cancer and melanoma), lesion volume and dichotomized survival with a cutoff value at 60 days were considered as covariates in a linear regression model with progression level as the independent variable and perfusion parameters the dependent variable. Two combinations of covariates improved the model, but only when correlating to rCBF: (1) melanoma and lesion volume and (2) age, melanoma, and lesion volume. Neither of the two linear models with covariates performed better than the other. Therefore, lesion volume and melanoma primary cancer were incorporated as covariates in the statistical evaluation of rCBF, since this model has fewer variables. While non-progressors with and without pseudo-progression are not easily distinguished from one another based on perfusion parameters, progressors exhibit lower rCBV and rCBF than non-progressors prior to SRS. Table 1 summarizes the results across enhancing tumor volumes, both including and excluding patients with no follow-up due to death.

**Table 1.** Comparisons of Relative Cerebral Blood Volume (rCBV) and Relative Cerebral Blood Flow (rCBF) Distributions in Patients Categorized as Non-Progressors and Progressors in Enhancing Metastatic Tumor Volume, Both Including and Excluding Patients With No Follow-up Due to Death

	Non-progression	Progression	*P*-value
rCBV [A.U.] (including non-follow-ups)	17.4 ± 7.0	8.5 ± 6.2	.002
rCBF [A.U.] (including non-follow-ups)	267 ± 123	132 ± 87	.01
rCBV [A.U.] (excluding non-follow-ups)	17.4 ± 7.0	11.2 ± 7.0	.08
rCBF [A.U.] (excluding non-follow-ups)	267 ± 123	167 ± 103	.21

Linear regression was performed with a significance threshold of 0.05.

rCBV estimated in the contrast-enhancing tumor volume in patients with short survival (less than 60 days after SRS) was significantly different from rCBV estimates in patients with longer survival (more than 60 days after SRS) (Table 2). rCBF, on the other hand, was not significantly different between short and long survivors. Only one out of six patients with short survival (less than 60 days after SRS) returned for a follow-up scan. No significant difference was found in survival when patients with pulmonary cancer were compared to the remaining patients.

**Table 2.** Comparisons of Relative Cerebral Blood Volume (rCBV) and Relative Cerebral Blood Flow (rCBF) Distributions in Patients Surviving Less Than 60 Days and Patients Surviving More Than 60 Days After Initial Stereotactic Radiosurgery in Enhancing Metastatic Tumor Volume

	<60 days	>60 days	*P*-value
rCBV [A.U.]	8.0 ± 6.6	15.8 ± 7.6	.03
rCBF [A.U.]	149 ± 131	238 ± 123	.14

Linear regression was performed, and a significance threshold of 0.05 was set.

The three patients receiving WBRT prior to SRS or craniotomy had lower rCBV (≥20% lower) at baseline than the average across patients, but higher than average rCBF (≥73% higher). No significant difference in rCBV and rCBF was found between patients receiving chemotherapy and those not receiving chemotherapy.

**Figure 4.**
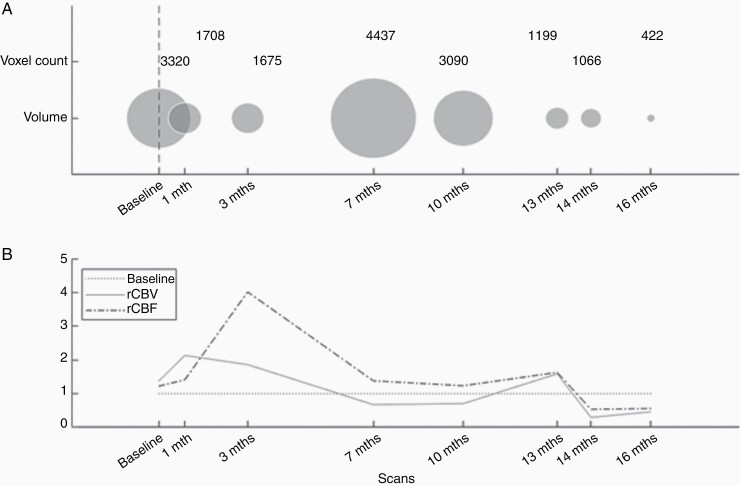
Transient rCBF increases prior to pseudo-progression in one patient. Development in time of lesion volume, relative cerebral blood volume (rCBV), and relative cerebral blood flow (rCBF) in a pseudo-progressor. The top panel shows the development of volume in time, along with a voxel-count of the total lesion volume. A vertical dashed line represents stereotactic radiosurgery. The bottom panel shows the development of rCBV and rCBF relative to the normalized baseline mean across all patients.

Despite small sample sizes, it is of interest that 5 of 7 pseudo-progressors display increasing rCBF values following SRS, yet prior to transient volume increase due to pseudo-progression (Figure 4). The same trend was seen in one additional patient, although rCBF did not decrease prior to the onset of the transient volume increase. Decreasing rCBF post-SRS was observed in the final patient. Among the patients that had a transient peak in rCBF post-treatment, prior to the transient volume increase, two had pulmonary metastases, one had colon metastasis, one had uterine metastasis and one had metastasis of uncertain origin (origo incerta). The two final pseudo-progressors had pulmonary metastases. A similarly consistent pattern of transient rCBF was not found in any of the patients with regressing and progressing metastases. In these 25 patients, only 10 patients returned for three or more follow-up scans.

## Discussion

By performing DSC-MRI perfusion acquisitions prior to and on the same day as SRS, it was investigated whether baseline perfusion levels could predict treatment outcome, ie, metastatic progression or non-progression, based on later lesion volume changes in time.

Patients experiencing progression exhibited significantly lower perfusion estimates in the enhancing metastatic tissue volume than the patients experiencing non-progression. Hypoxic tumors are less radiation sensitive to conventional radiation therapy and SRS,^44,45^ offering a plausible explanation for the low perfusion estimates in patients with subsequent disease progression. Short survival was not a factor that improved the linear regression models, however, when testing if there was a significant difference in perfusion parameters in short survivors, rCBV was significantly lower in short survivors. rCBF on the other hand, was not significantly different in short and long survivors, although patients with progressing disease may be more likely to exhibit lower rCBF. Based on our data, it is not possible to distinguish patients experiencing non-progression from patients experiencing non-progression with pseudo-progression. This means that poor initial vascularity does not necessarily mean that the patient will not benefit from SRS, but if a volume increase is detected at a later point in time, it could be an argument for suspecting true disease progression (and not pseudo-progression), which in turn could alert treatment decisions. However, these propositions must be regarded as possible hypotheses. Our results are inconclusive in this regard, since when removing patients without follow-up from the study, we no longer report significant findings. Low perfusion estimates seem to be connected to an unfavorable outcome, due to both disease progression and poor survival.

In the literature, rCBV is reported more commonly than rCBF. Typically, significantly higher rCBV values are reported in recurrent metastatic tumors compared to radiation necrosis in progressively enhancing tumors.^22,24,27–29^ But literature is not unanimous in this matter, as lower rCBV at 1-month follow-up has also been reported.^20^ Furthermore, these studies report on measurements that are taken after treatment.^22,24,27–29^ Treatment responders (ie, non-progressors) have previously been observed to have increased rCBV at baseline,^33^ but pretherapeutic rCBV and rCBF are not always observed to correlate with treatment outcome.^20,32,35^ Our findings are in agreement with this. Decreased rCBF at 6-week follow-up has previously been found to indicate treatment response.^32^ As pseudo-progression does not necessarily take place at a set interval of weeks following treatments, we simply report a transient increase in rCBF following SRS, prior to a transient volume increase in 5 out of 7 non-progressors with pseudo-progression. This may suggest that pseudo-progressors have a more active microvasculature than regressors or progressors. We found no similar pattern in progressors and regressors, but it should be noted that most of these patients returned for few follow-up visits or underwent an immediate second SRS treatment. Since 17 out of 32 patients had pulmonary metastases, our data do not support that pulmonary metastasis is tied to the response. Comparisons across studies are difficult, because like ours, the number of patients included in the studies is small, usually based on a retrospective design and patient prognosis based on transient volume changes as a proxy of disease progression instead of histopathology.

Patients undergoing SRS have a metal frame pinned to their skulls prior to MR imaging, as a precise localization of the lesion relative to the gamma knife frame of reference is of paramount importance, particularly since high radiation doses are administered. In the current study, imaging was performed prior to fixation of the metal frame to avoid susceptibility artifacts in the rapidly acquiring data collection of the echo-planar imaging sequence. The frame was later pinned to the skull, and a final 3D volume post-contrast was acquired based on the already administered contrast. It is not clear how this was handled in previous studies, and difference in procedures could contribute to the differences reported in findings in the literature as well as many sites omitting acquiring baseline data. This is probably one of the reasons why most perfusion studies in SRS report only follow-up data.

Tracer kinetic modeling in DSC-MRI assumes that the blood-brain barrier is intact, so that contrast agent does not leak into the extravascular-extracellular space during the acquisition time. Such a leakage would cause additional signal attenuation due to magnetization dispersion effects in the extravascular-extracellular space but was corrected for in the current study through modeling.

Limitations of the study include a small number of participants, lack of follow-up imaging due to early death in five out of the 41 patients, lack of histopathological confirmation of progression vs pseudo-progression, and heterogeneity of the study cohort both in terms of primary cancers and in terms of additional treatment. However, the inclusion of patients that received WBRT or craniotomy did not increase measured rCBV in non-progressors or long survivors and can therefore not explain high rCBV in non-progressors or long survivors.

Data were acquired using 2D acquisition of perfusion data with inherent limited spatial coverage. This could potentially be improved if data were acquired at a 3 T instead of a 1.5 T, however, both artifacts and contrast agent non-linearity effects are also augmented at higher field strength. There is always an inherent trade-off between increased spatial resolution and the need for high temporal resolution in DSC-MRI.

In conclusion, lower perfusion estimated prior to SRS may be associated with disease that progresses after SRS, though results should be verified in different or larger patient material. This suggests the hypothesis that interpretation of increasing enhanced tumor volume post-SRS may be helped by early DSC-MRI parameter estimates, which in turn could influence treatment decisions. A transient increase in 5 out of 7 non-progressors with pseudo-progression was observed.
